# Therapeutic Features and Updated Clinical Trials of Mesenchymal Stem Cell (MSC)-Derived Exosomes

**DOI:** 10.3390/jcm10040711

**Published:** 2021-02-11

**Authors:** Byung-Chul Lee, Insung Kang, Kyung-Rok Yu

**Affiliations:** 1 Translational Stem Cell Biology Branch, National Heart, Lung, and Blood Institute, NIH, Bethesda, MD 20892, USA; byung-chul.lee@nih.gov; 2 Program in Developmental Endocrinology and Genetics, Eunice Kennedy Shriver National Institute of Child Health and Human Development, NIH, Bethesda, MD 20892, USA; insung.kang@nih.gov; 3 Department of Agricultural Biotechnology and Research Institute of Agriculture and Life Sciences, Seoul National University, Seoul 08826, Korea

**Keywords:** exosome therapy, clinical trials, immunomodulation, regeneration, angiogenesis

## Abstract

Identification of the immunomodulatory and regenerative properties of mesenchymal stem cells (MSCs) have made them an attractive alternative therapeutic option for diseases with no effective treatment options. Numerous clinical trials have followed; however, issues such as infusional toxicity and cellular rejection have been reported. To address these problems associated with cell-based therapy, MSC exosome therapy was developed and has shown promising clinical outcomes. MSC exosomes are nanosized vesicles secreted from MSCs and represent a non-cellular therapeutic agent. MSC exosomes retain therapeutic features of the cells from which they originated including genetic material, lipids, and proteins. Similar to MSCs, exosomes can induce cell differentiation, immunoregulation, angiogenesis, and tumor suppression. MSC exosomes have therefore been employed in several experimental models and clinical studies. Here, we review the therapeutic potential of MSC-derived exosomes and summarize currently ongoing clinical trials according to disease type. In addition, we propose several functional enhancement strategies for the effective clinical application of MSC exosome therapy.

## 1. Background

Mesenchymal stem cells (MSCs) have unique biomedical properties due to their stemness: they can simulate their own proliferation and can differentiate into multi-lineage cells [1]. MSCs have low immunogenicity due to low expression of major histocompatibility complex (MHC) class I molecules and expression of only a few MHC class II molecules [2,3]. Importantly, MSCs have been demonstrated to have immunomodulatory and regenerative functions in a variety of disease models [4,5,6,7,8]. Given these properties, MSCs have been evaluated in clinical trials over several decades [9]. Even though the results of some preclinical studies and clinical trials using MSCs were promising, clinical outcomes often did not improve significantly or disease severity increased after MSC therapy in other studies, making MSC-based therapy controversial [10]. These discrepancies in the effects of MSCs could be due to poor engraftment of infused MSCs [11,12], donor-dependent variation, or possible infusional toxicity due to lodging of cells in the microvasculature [13,14]. Moreover, senescence of MSCs during in vitro expansion makes the cells less productive, and can increase disease severity by causing inflammaging, which refers to a persistent, low-grade, systemic pro-inflammatory status that is characteristic of the normal aging process of mammalian tissues and cells [15].

To guarantee consistent and stable therapeutic effects, improved MSC therapeutic strategies are needed [16]. Strategies should take into account current hurdles such as low in vivo survival, inaccurate delivery to inflamed lesions, and donor-to-donor variation. Researchers have attempted to enhance the therapeutic efficacy of MSCs by improving their migration into lesions [17,18] and by preconditioning them with bioactive molecules such as IFN-γ and TNF-α [19,20]. Furthermore, use of co-administration strategies including scaffolds and hydrogels have enabled more deliberate and accurate disease treatment. An alternative approach to improve MSC-based therapy is to use membrane-bound extracellular vesicles (EVs) or exosomes [21].

From prokaryotes to eukaryotes, released EVs are involved in intercellular communication by transmitting biological signals between cells. Based on their biogenesis, EVs are categorized into three main classes: exosomes, microvesicles, and apoptotic bodies [22]. With the smallest size range of around 50 to 150 nm, exosomes are released into the extracellular environment when multivesicular bodies fuse with the cell membrane. These nanometer-sized vesicles contain many constituents of cells, including cytokines and growth factors, signaling lipids, and mRNA and regulatory miRNAs, with variation in the components of the exosome according to their cellular origin. Notably, MSC exosomes may be more suitable for clinical application than MSCs. MSC exosomes are likely to be free of immunogenic problems and unlikely to be trapped in the lung or liver like infused MSCs, while still maintaining the therapeutic functions of their cells of origin. Given the proven therapeutic potential of MSC-derived exosomes in preclinical studies, there are currently 16 ongoing clinical studies investigating the therapeutic application of MSC-derived exosomes in various diseases [23].

In the present study, we provide a comprehensive review of the therapeutic potential of exosomes from various MSC sources and their clinical applications based on the 16 clinical trials registered in a public database by discussing their effects in a disease-specific manner.

## 2. Therapeutic Properties of MSC Exosomes

**Figure 1:**
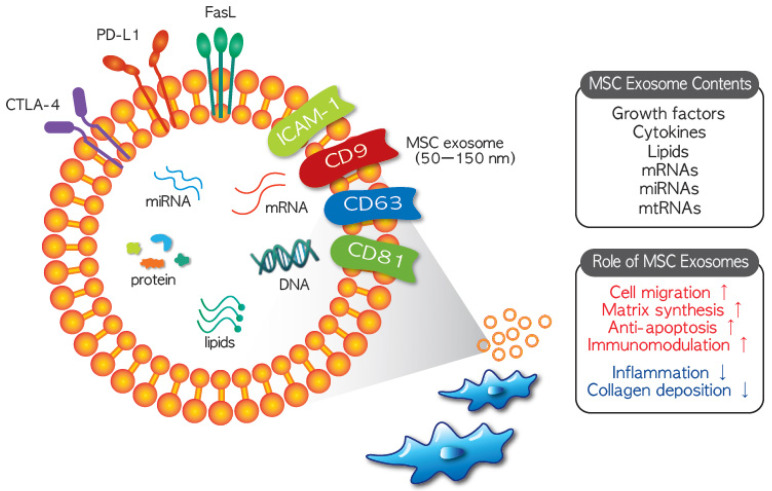
Composition and therapeutic function of MSC (mesenchymal stem cells) exosomes. Exosomes derived from MSCs may contain growth factors, cytokines, DNA, lipids, mRNAs, miRNAs, and mtRNAs. Similar as their cells from which they originated, MSC exosomes possess therapeutic properties including stimulation of cell migration and extracellular matrix synthesis, anti-apoptotic effects, immunomodulation and anti-inflammatory effects, and stimulation of collagen deposition. Due to these distinctive therapeutic features, the feasibility of MSC exosome therapy is currently being assessed in several clinical studies.

Mesenchymal stem cells have been evaluated as alternative therapies for a variety of rare diseases due to their ability to immunomodulate activated immune cells and stimulate tissue regeneration. Two main cellular behaviors underlie their therapeutic effects: direct cell-mediated action and environmental changes induced by release of soluble factors [24]. The MSC secretome, which refers to soluble factors released into the culture medium, has recently attracted attention. Among the released substances, microvesicles (MVs, 0.1–1 mm in diameter) and exosomes (50–150 nm in diameter) function as intercellular mediators between MSCs and target cells, which include MSCs [25]. Nanosized exosomes (Figure 1) in particular are considered MSC substitutes because they have similar therapeutic features to MSCs. Exosomes may have greater potential for clinical application than MSCs because of their low immunogenic and tumorigenic potential [26]. Thus, the use of MSC exosomes has opened up new avenues for clinical application of MSCs.

The most important property of MSC-derived exosomes is their ‘cargo’ function; they contain a variety of validated therapeutic agents. Contents of MSC exosomes include nucleic acids, proteins, and lipids, with more than 850 gene products and 150 miRNAs described [27]. Moreover, their composition can vary depending on the source the MSCs were isolated from and the external stimuli, which is one of the reasons why MSCs from different sources exhibit different characteristics or therapeutic effects [26]. Although the characteristics of exosomes from different MSC sources can vary, they are in general therapeutic. MSC exosomes horizontally transfer their contents such as mRNAs, miRNAs, and proteins into target cells to modify the cellular functions of these cells [28]. Although the mechanisms of MSC exosome endocytosis have not yet been fully elucidated, several cellular processes including vesicle-cell fusion, phagocytosis, micropinocytosis, and/or receptor-mediated endocytosis are assumed to be involved in internalization [29]. However, some researchers have claimed that internalization of MSC exosome into target cells is not necessary for them to exert a therapeutic effect [30,31].

Several clinical features of MSC exosomes have been described in preclinical studies. For example, MSC exosomes accelerate the tissue regeneration process by regulating proliferation and apoptosis of endogenous cells [32,33,34]. MSC exosomes also affect the fate decision of some immune cells, thus facilitating attenuation of excessive inflammation or a return to immune homeostasis [35,36,37]. Furthermore, MSC exosomes induce angiogenesis by stimulating various cellular signaling pathways [38,39,40]. Given these promising results, safety and efficacy of application of MSC exosomes are currently being evaluated in clinical trials targeting various diseases. Below, we review the therapeutic potential of MSC exosomes according to disease type.

## 3. Immune-Related Diseases

The most important clinical feature of MSCs and MSC-derived exosomes is their immunomodulatory function [41]. Immune-related diseases are generally due to imbalances in the immune system and subsequent responses of the body against biased activation of specific types of immune responses. MSC exosomes have attracted interest as major mediators of MSC-mediated immunomodulation of various types of immune cells. Exosomes derived from MSCs play a role in regulating various immune cell subsets and subsequently facilitating immune homeostasis, like their cells of origin. MSC exosomes have been shown to have suppressive functions against activated immune cells including effector T cells, microglial cells [42], macrophages [43], and NK cells [44]. In contrast, MSC exosomes facilitate lineage determination toward regulatory type immune cells such as tolerogenic dendritic cells [37], M2 macrophages [45], and regulatory T cells [42], promoting immune system homeostasis. The modulatory functions proven in preclinical studies have inspired clinical trials targeting various autoimmune diseases.

MSC exosomes efficiently suppress excessive proliferation and activation of Th1 cells. For example, CD39-expressing CD4^+^ Th1 cells initiated adenosine-related apoptosis after internalizing BM-MSC derived exosomes in an animal graft-versus-host disease (GvHD) model [46]. Furthermore, the therapeutic function against GvHD has demonstrated in human study [47]. According to a previous study using a contact hypersensitivity (CHS) model mouse, infusion with exosomes from umbilical cord-derived MSCs (UC-MSCs) resulted in a decreased level of Th1 cells and subsequent secretion of IFN-γ with increased Treg induction. Internalization of EVs was also shown to be associated with decreased expression of signal transducer and activator of transcription 1 (STAT1), which has a crucial role in Th1 cell development [48]. Although simultaneous increases in Th17 and Treg by treatment with MSC exosomes have been reported [49], Th17 cells and Tregs are generally considered to be in a competitive relationship. Indeed, MSC exosomes have been reported to promote Tregs while suppressing the polarization of T_H_17 cells by SphK1-mediated SP1 enrichment in an aplastic anemia (AA) model [50] and increase miR-125a and miR-125b expression in an experimental colitis model [51]. MSC exosomes also suppress excessive allergic inflammation. Cho et al. reported that infusion of exosomes derived from adipose tissue-derived MSCs (AT-MSCs) alleviated atopic dermatitis (AD) by improving pathological symptoms and infiltration of mast cells into skin lesions [52]. Asthma, a major allergic disease of the airways, was controlled by MSC EV’s infusion due to suppression of group 2 innate lymphoid cell (ILC2) pathways [53] or promotion of the Treg population [54]. Given these preclinical findings, the clinical potential of MSC exosomes is currently being assessed in four different immune disorders (Table 1)

Approximately 35–50% of patients suffer from GvHD after allogeneic hematopoietic stem cell (HSC) transplantation, which is caused by donated hematopoietic stem cells perceiving recipient body systems as foreign and attacking them. Chronic GvHD (cGvHD) is characterized by rashes, abdominal swelling, dry eyes, vision changes, shortness of breath, muscle weakness, and tightness in joints [55,56]. Among these symptoms, dry eye is the most frequent, occurring in 40–76% of patients, and its severity could reflect disease prognosis. Keratoconjunctivitis sicca is caused by lymphocytic infiltration around lacrimal glands, resulting in tissue degeneration and malfunction [57]. Since immunosuppressants such as corticosteroids or cyclosporine A nonspecifically suppress the immune system and are not appropriate in all patients, MSCs have been considered as an alternative therapy to treat dry eye in cGvHD [58]. A clinical trial evaluating the therapeutic efficacy of umbilical MSC (UMSC) exosomes at alleviating dry eye symptom was registered in December 2019 (NCT04213248). Notably, the aims of this trial are to analyze the responses of disease indicators, such as the amount of tears and the degree of damage to tissues, to exosome treatment with the exosomes applied in the form of eye drops rather than intravascular injection.

Type 1 diabetes mellitus (T1DM) is another well-known autoimmune disease that needs life-long management. Symptoms include weight loss, exhaustion, itchy skin, and blurry eyesight. Immune cells in the body attack pancreatic β cells for unknown reasons, adversely affecting insulin production and subsequent glucose metabolism. As there is as yet no specific treatment, patients with T1DM require daily insulin injections or installation of an insulin pump to maintain a normal range of blood glucose levels [59,60]. To address the absence of drugs specific for treating T1DM, MSC exosomes have been explored. Nojehdehi et al. reported that exosomes derived from AD-MSCs had a therapeutic effect in a streptozotocin-induced T1DM mouse model by increasing the Treg population in the spleen and regenerating pancreatic islets [61]. The exosome from menstrual MSCs has shown similar therapeutic potential in a rat model, potentially mediated by a pancreatic and duodenal homeobox 1 pathway [62]. A clinical trial evaluating the effects of repetitive intravenous infusions of UCB blood-derived MSC exosomes in T1DM is currently underway (NCT02138331).

In addition to these autoimmune diseases, MSC exosomes have been shown to alleviate experimental periodontitis. Dysbiosis in the periodontal cavity promotes the growth of pathogenic microbiota such as *Porphyromonas gingivalis* and *Fusobacterium nucleatum*, contributing to autoantibody generation and subversion of host immune responses after invasion of these autoantibodies into the circulatory system and various organs [63]. Xu et al. demonstrated that exosomes from P2 × 7 receptor (P2 × 7R) gene-modified periodontal ligament stem cells (PDLSCs) restored the compromised regenerative function of neighboring cells by binding to the GREM-1 protein and upregulating the expression of miRNAs including miR-3679-5p, miR-6515-5p, and miR-6747-5p [64]. Exosomal microRNA-155-5p from PDLSCs directly rectified the imbalance in the Th17/Treg ratio by regulating the expression of sirtuin-1 (SIRT1) in a chronic periodontitis experimental model [65]. Moreover, adipose tissue-derived MSC (AD-MSC) exosomes have been reported to play a supportive role in periodontal ligament and tissue regeneration [66]. In a clinical study, the therapeutic efficacy of autologous administration of AD-MSC exosomes at alleviating gingival inflammation and tissue damage in patients aged between 18 to 50 years is being evaluated (NCT04270006). Lastly, multiple organ dysfunction syndrome (MODS), a death-causing postoperative complication after cardiovascular surgery [67], is being treated with exosomes isolated from UC-MSCs (NCT04356300). The proinflammatory cytokine, IL-6, is often employed as a prognostic indicator of MODS, because IL-6 is released in response to tissue injury and inflammatory stimuli [68,69]. To assess the safety and efficacy of MSC exosomes, short- and long-term immune responses, concentration of IL-6 (early 3 days), and allergic reactions including rashes, itching, and anaphylactic shock (up to 6 months) as well as organ failure assessment scores are being investigated.

## 4. Wound Healing

Exosomes from various MSCs have been shown to accelerate wound repair [70,71,72,73]. Even though wound healing is a complicated physiological phenomenon, MSC exosomes contribute to the recovery process by promoting proliferation, differentiation, positioning of cellular constituents of skin [74], and inducing the differentiation of immune cells [45]. Subcutaneous administration of exosomes derived from iPSC-MSCs has been shown to accelerate wound repair in an animal model by promoting type I and III collagen and elastin secretion by fibroblasts [71]. In addition, transplanted MSC exosomes play a crucial role in not only angiogenesis but maturation of vessels in wound sites to boost the healing process [71,72].

Epidermolysis bullosa (EB) is a disease characterized by skin fragility and blistering in response to mechanical trauma with 30 distinct subtypes [75]. Among disease subtypes, recessive dystrophic epidermolysis bullosa (RDEB) resulting from mutations in the type VII collagen (*COL7A1*) gene and subsequent lack of protein production can be alleviated by bone marrow-derived MSC (BM-MSC) EVs because they donate their own type VII collagen and induce host fibroblasts to produce this protein [76]. Given these promising results, a phase I/IIA clinical study was launched to test the safety and feasibility of a topical application of allogeneic MSC exosomes in EB. Dose-limiting toxicity and wound size recession will be assessed in 10 participants (NCT04173650). Moreover, another prospective clinical study using BM-MSCs has reported that intravenous infusion was well tolerated with no severe complications up to 12 months and that there was transient alleviation of EB symptoms for 2 months (NCT02323789). In this study, the authors emphasized the relevance of the secretome, including EVs and exosome, in tissue regeneration [77].

Besides skin and epidermal wounds, MSC exosomes have been also applied to wound healing of other organs. Idiopathic macular hole (MH) can induce retinal detachment in highly myopic eyes [78]. Although surgical management via pars plana vitrectomy (PPV) is used to treat this condition in the majority of the patients, those patients with particularly large and long-lasting MHs have a poor prognosis after standard PPV surgery [79]. Alternative or auxiliary tools to promote functional and physical recovery from MHs have therefore been explored. Zhang et al. reported that six of seven patients (two patients treated with MSC exosomes and five patients treated with umbilical cord-derived MSC exosomes by intravitreal injection after surgical procedures) showed complete macular closure while five patients showed improved visual function as assessed by best corrected visual acuity (BCVA) [79], the primary outcome measure of the clinical study (NCT03437759).

## 5. Neurological Diseases

The focus in stem cell therapy has shifted from direct cell-to-cell interactions to paracrine interactions after acknowledging that transplanted cells migrate poorly to existing neural networks and that their therapeutic effect is orchestrated by secretion of heterogeneous EVs [80]. Moreover, advances in modifying molecules to enable them to cross the blood-brain barrier (BBB) has brought exosomes in the spotlight as therapeutic candidates for neurological diseases and mental disorders. Alvarex-Erviti et al. developed neuron-targeting exosomes loaded with *GAPDH* siRNA [81]. Injection of these exosomes intravenously resulted in specific gene knockdown in neuronal cells [81]. Given the evidence that exosomes can cross the BBB, Xin et al. administered rat bone marrow-derived MSC exosomes containing miR-17-92 to a stroke model via an intravenous route and demonstrated enhancement of oligodendrogenesis, neurogenesis, and neuroplasticity with functional recovery [82]. Recently, intravenously administrated MSC exosomes have been modified to target specific regions of the brain. Cui et al. modified exosomes with the central nervous system-specific rabies viral glycoprotein (RVG) peptide to target them to the cortex and hippocampus in an Alzheimer’s disease mouse model. RVG-tagged MSC exosomes reduced plaque accumulation and astrocyte activation and decreased expression of the pro-inflammatory mediators TNF-α, IL-β, and IL-6 and increased levels of the anti-inflammatory factors IL-10, IL-4, and IL-13 [83].

Another direct pathway to the brain is the nasal cavity; this route can be used to bypass the BBB to deliver therapeutic agents to the brain [84]. In a pilocarpine-induced status epilepticus mice model, MSC-derived exosomes were administrated intranasally and were reported to reach the hippocampus within 6 h, where they had neuroprotective and anti-inflammatory effects [85]. Perets et al. evaluated the effects of MSC-exosome intranasal administration in BTBR T+tf/J (BTBR) mice, an accepted model of autistic-like behavior, and reported increased male-male social interaction with reduced repetitive behavior and improvement in maternal behavior, suggesting a therapeutic strategy to reduce symptoms associated with autism spectrum disorders [86]. Guo et al. were able to detect MSC exosomes in spinal cord lesions of a spinal cord injury model after intranasal delivery. Specifically, phosphatase and tensin homolog small interfering RNA (ExoPTEN)-loaded exosomes reduced the expression of PTEN, thereby improving structural and electrophysiological function in spinal cord injury.

Given that exosomes delivered intravenously can cross the BBB, the Jordan group from Neurological Associates of West Los Angeles has initiated two separate trials targeting craniofacial neuralgia (NCT04202783) and neurodegenerative disease-driven depression, anxiety, and dementia (NCT04202770). The Wang group from Ruijin Hospital in China has initiated a phase I/Ⅱ clinical trial to explore the safety and efficacy of exosomes derived from allogeneic adipose tissue derived-MSCs for the treatment of mild to moderate dementia due to Alzheimer’s disease (NCT04388982). They plan to administer exosomes at three different doses twice a week for 12 weeks and will explore the safety and efficiency of these exosomes and provide a clinical dose reference for subsequent trials.

## 6. Cardiovascular Diseases

Although mortality rates have declined dramatically over the past two decades, cardiovascular and circulatory diseases are still recognized as the leading causes of death worldwide [87]. While the primary cause of death in the United States is cardiovascular disease [88], ischemic heart disease, a major cause of cardiovascular disease, leads the death rate in China [89]. Due to cardiomyocyte loss in ischemic heart diseases, investigators have focused on the importance of regenerative medicine to prevent cardiovascular disease. The ability of MSCs to differentiate into a variety of cell types has led to investigation of MSCs as major cell-based therapeutic agents for cardiac tissue regeneration and repair.

Although MSC stem cell therapy results have been promising, it is still unclear how they work. Freyman et al. observed only 30,000 cells from 50 × 10^6^ engrafted cells injected intravenously, representing 0.06% of the population, in the infarct zones of swine heart after acute myocardial infarction [90]. Timmers et al. showed that MSC-conditioned media treatment after myocardial infarction in swine preserved cardiac function, suggesting that MSC secretions may have angiogenic potential [91]. Shao et al. compared MSC-derived exosome-treated and stem cell-treated rat myocardial infarction models and reported that MSC exosomes inhibited cardiac fibrosis and inflammation and improved cardiac function to a greater extent than MSCs [92].

Given that the function of secretory exosomes is determined by the materials they contain such as cytokines, proteins, mRNAs, miRNAs, and rRNAs, studies have been performed to identify key factors involved in cardiac regeneration. Anderson et al. identified 1927 proteins in MSC-derived exosomes and analyzed nuclear factor-kappaB; signaling as a key mediator of MSC-induced angiogenesis [38]. In rat myocardial infarction model, Wang et al. showed that the cardioprotective effect of MSC exosomes was mediated by miR-21 enhanced cell survival via the PTEN/Akt pathway [93]. A recent study reported that MSC exosomes containing miR-25-3p had cardioprotective effects by decreasing EZH2, H2K27me3, and SOCS3 expression, alleviating myocardial infarction by targeting pro-apoptotic proteins and inflammatory genes [94]. According to the finding that MSC-derived exosomes promote angiogenesis remodeling and functional recovery after stroke, a recent clinical study form Isfahan University of Medical Sciences is exploring the use of miR-124-loaded MSC-derived exosomes to improve angiogenesis in patients with acute stroke (NCT03384433).

## 7. Cancers

The application of MSC-derived exosomes in cancer therapy has been investigated for several years. Although there are some controversies on the functions on tumor progression, several reports showed the inhibition effect of MSC EVs in tumor metastasis. Patients in recession or those who are undergoing chemotherapy occasionally experience cancer recurrence due to undetectable and slow-growing dormant cells. In 2014, Ono et al. reported that the BM-MSC exosome can inhibit metastatic cancer cell proliferation and consequently promote dormancy in metastatic breast cancer cells through miR23b-mediated suppression of MARCKS [95]. Moreover, cancer cell priming was found to be essential for the anti-cancer effect of the MSC exosome by promoting cycling quiescence in cancer cells [96,97]. MSC exosomes can also mediate changes in drug resistance development by gastric cancer cells via calcium/calmodulin-dependent protein kinase (CaM-K) and Raf/MEK/ERK kinase cascade signaling, which would improve the efficiency of chemotherapy [98]. In contrast to their effects in cardiovascular disease, MSC-derived exosomes function as intercellular mediators between tumor cells and suppress angiogenesis by directly transferring anti-angiogenic molecules such as miR-16 and suppressing VEGF expression [99].

However, Lin et al. revealed that MSC exosomes accelerate migration of cancer cells and expression of cancer-related pathways, including the Wnt signaling pathway [100]. Similarly, MSC exosomes were shown to induce the epithelial-mesenchymal transition (EMT) in gastric cancer cells and enhance tumorigenicity by upregulating cancer growth and migration [101]. Their angiogenic properties are also of concern in the context of promoting cancer growth. Thus, MSC exosome-cancer therapy is sometimes referred to as a “double-edged sword” [102,103]. To overcome this, researchers have focused on the cargo function of MSC exosomes. For example, exosomes derived from MSCs treated with taxol (paclitaxel; PTX) showed improved tumor-suppressive effects, resulting in direct tumor cell growth inhibition at a 1000-fold decreased concentration compared to taxol mono-treatment [104,105]. Moreover, siRNA targeting tumor-related genes such as polo-like kinase-1 (PLK-1) can be loaded within MSC-derived exosomes for intravesical therapy [106].

Accordingly, MSC exosomes containing siRNA targeting oncogenic Kras^G12D^ mutations are being employed against pancreatic cancer in a clinical trial (NCT03608631). The Kras^G12D^ mutation, which is a common mutation in pancreatic cancer, is a promising therapeutic target. Kalluri group has developed engineered exosomes (iExosomes) expressing CD47 and si- or sh-RNA for Kras^G12D^. They demonstrated that iExosomes suppressed oncogenic Kras and enhanced the survival of pancreatic cancer mouse model [107,108]. Based on these findings, the safety and feasibility of iExosomes need to be evaluated in patients with Kras^G12D^-related pancreatic cancer in clinical trials.

## 8. MSC Exosomes as a COVID-19 Supportive Treatment

Recently, coronavirus disease 2019 (COVID-19), caused by rapid and widespread infection by the novel coronavirus severe acute respiratory syndrome coronavirus 2 (SARS-CoV-2), has become the biggest general threat to both global public health and the world economy. Accordingly, pragmatic methods to prevent and treat infection are greatly in demand [109,110,111,112,113,114].

Similar to other coronaviruses such as severe acute respiratory syndrome (SARS-CoV) and Middle East respiratory syndrome (MERS-CoV), SARS-CoV-2 primarily targets the human respiratory system and symptoms include fever, dry cough, sputum, fatigue, headache, and dyspnea. Importantly, this virus affects the lower respiratory tract resulting in infiltration of the upper lobes of the lungs and subsequent hypoxemia, unlike other coronaviruses. In addition, patients infected by SARS-CoV-2 occasionally present with diarrhea as one of the symptoms [115]. SARS-CoV-2 binds to the angiotensin-converting enzyme 2 (ACE2) receptor present on host lung cells using its S protein and once internalized, begins to replicate itself [116]. Although CD4^+^ and CD8^+^ T cells expressing HLA-DR and CD38, respectively, remain activated, the number of these cells in the peripheral blood of patients with COVID-19 is reportedly reduced [117]. SARS-CoV-2 infection disrupts the function of a patient’s immune system. Dysregulated immune effector cells secrete large amounts of pro-inflammatory cytokines (e.g., IFN-α, IFN-γ, IL-1β, IL-6, IL-12, IL-18, IL-33, TNF-α, TGFβ) and chemokines (e.g., CCL2, CCL3, CCL5, CXCL8, CXCL9, CXCL10), leading to a so-called “cytokine storm” [118]. Increased cytokine secretion and subsequent infiltration of the lung tissue by activated immune cells such as inflammatory monocytes and neutrophils triggers acute respiratory distress syndrome (ARDS), which is widely recognized as one of the most critical factors resulting in death from COVID-19 [119]. Thus, an effective treatment to prevent a COVID-19-induced cytokine storm is urgently needed.

As we described above, the most prominent feature of MSCs or exosomes derived from MSCs is their immunomodulation ability. MSC exosomes or EVs have already been employed to treat airway diseases such as asthma [54], bronchopulmonary dysplasia [43,120], and ARDS [121]. MSCs and their derivatives have also been shown to efficiently suppress influenza virus infection by mitigating symptoms and restoring normal physiological function. In addition, clinical trials have demonstrated that MSC transplantation reduces the severity of influenza virus-induced lung injuries and lowers mortality, suggesting a potential role for MSCs and MSC EVs in COVID-19 treatment [122,123,124]. Even though the production of exosomes is technically and economically inefficient compared to MSCs themselves, exosomes have advantages for treating airway-borne diseases because exosomes are not trapped in the lungs unlike MSCs injected via the intravenous route. Moreover, with a size of around 100 nm, exosomes can be applied via aerosol inhalation while preserving their immunomodulatory function [125]. Due to their unique phospholipid membrane structure, bioactive molecules, including promising drugs, can be inserted into exosomes and their effects could be maximized by fusing target cell membranes and transferring the cargo into the cytoplasm [126].

Currently, there are four clinical trials exploring the use of MSC exosomes as a supportive treatment for COVID-19-infected patients. The first case entitled ‘A Pilot Clinical Study on Inhalation of Mesenchymal Stem Cells Exosomes Treating Severe Novel Coronavirus Pneumonia’ was designed to explore the therapeutic potential of aerosol inhalation of exosomes derived from allogenic AT-MSCs in patients with severe disease (NCT04276987). The safety of this MSC exosome treatment is being assessed in a separate clinical study (NCT04313647). Another study led by Russian clinicians is testing whether inhalation of MSC-derived exosomes can suppress over-response of the immune system to the virus and stimulate regenerative processes (NCT04491240, NCT04602442). Lastly, even though an intravenous route will be employed for actual injection, administration of EVs derived from BM-MSCs or ‘ExoFlo’ is being evaluated as a treatment for moderate-to-severe ARDS in patients with COVID-19 (NCT04493242) [127]. In addition to these three studies, studies using EVs or exosomes from non-MSC sources including donor-originated COVID-19 specific T-cells (NCT04389385) or amniotic stem and epithelial cells with over 300 growth factors, cytokines, and chemokines derived from human amniotic fluid (HAF) (NCT04384445) are also being performed (Table 1). Even if these ongoing studies report successful outcomes, there are still several issues that need to be resolved. Of note, safety issues should be addressed before actual clinical application, because MSC EVs reportedly have procoagulant activity as do their cells of origin [128]. A cohort study revealed that severe COVID-19 patients present with hypercoagulability as one of their symptoms [129]; thus, procoagulant factors as well as excessive proinflammatory cytokines in the exosome cargo must be removed prior to infusion as recommended in plasma exchange therapy [130]. Moreover, to expedite and consolidate preclinical processes, well-established disease models such as a nonhuman primate models are needed to assess the safety and efficacy of novel therapeutics and potential vaccines.

## 9. Future Perspectives and Concluding Remarks

**Figure 2:**
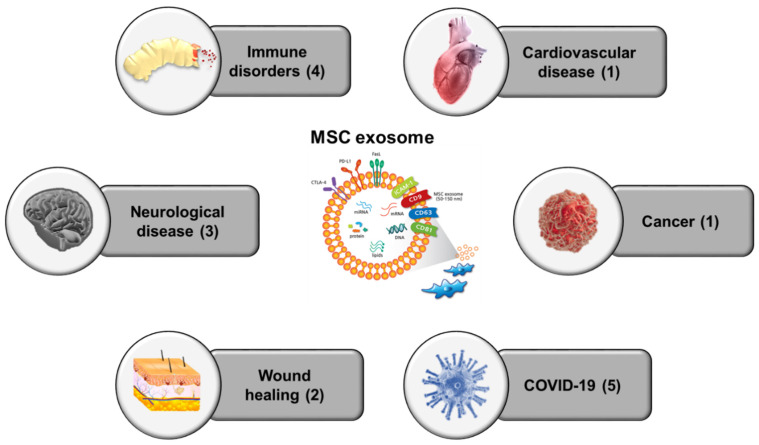
Current clinical trials of MSC exosome therapy. Summarized figure of 16 registered clinical trials categorizing by disease type. The numbers in the parentheses indicated ongoing clinical studies (~Jan 2021).

In the present study, we comprehensively reviewed the therapeutic features and current status of clinical trials of MSC exosomes according to disease type (Figure 2). Although preclinical studies have reported promising results, several enhancements of MSC exosome therapy are required to obtain superior outcomes. First, the suitability of MSCs for application to specific diseases should be evaluated prior to exosome isolation. Genetic modification of MSCs can improve the therapeutic function of exosomes derived from these MSCs. One of the primary targets of genetic modification could be miRNAs. For example, exosomes derived from MSCs overexpressing miR-92a-3p facilitated superior chondrogenesis and cartilage protection compared to naïve cells [131] and the bone regenerative function of AD-MSC exosomes was shown to be enhanced by miR-375 overexpression [132]. Moreover, overexpression of miR-143 improved the suppressive function of MSC exosomes on cancer cell migration and invasion [133]. There are numerous other miRNAs that are potential targets for genetic manipulation in exosomes in addition to the miRNAs discussed above. Overexpression of proteins with specific therapeutic roles such as CXCR4 [134] and GATA-4 [135] has also been demonstrated to improve the function of MSC exosomes. Thus, prior to genetic manipulation of MSCs, it is important to identify precise genetic targets that can be manipulated in exosomes to treat specific diseases.

Second, the therapeutic effect of MSC exosomes is reinforced by direct delivery of target molecules into MSC exosomes. A variety of insertion methods including passive loading, sonication, electroporation, extrusion, and light-induced loading have already been developed [136]. As we discussed above, anticancer drugs have been loaded into exosomes to achieve better clinical outcomes [104,105]. Sun et al. previously reported that the anti-inflammatory activity of curcumin, a potent immune suppressant, was improved by encapsulating it in exosomes [137]. Another anti-inflammatory drug, namely the STAT3 inhibitor JSI124, efficiently suppressed neuroinflammation when introduced in the form of exosomes via the intranasal route [138]. In addition to exosome-mediated drug delivery, loading and delivery of long endogenous RNAs or small interfering RNAs has been shown to inhibit cancers and results in apoptosis of leukemic cells [139,140].

Supportive materials can be utilized to maximize the therapeutic functions of MSC exosomes. Among biocompatible aids, various hydrogels have recently attracted attention. Placenta-derived MSC exosomes containing chitosan hydrogel showed enhanced therapeutic effectiveness with retention in vivo and stability of exosome contents, including proteins and miRNAs, in an ischemic experimental model [141]. Other hydrogels have been shown to strengthen the wound repair process by complementing physical defects [73,142]. In a similar context, packing of tissues with MSC exosomes and scaffolds resulted in better outcomes than naïve treatment in bone or endometrium regeneration models [143,144,145]. Additionally, pretreatment of MSCs with bacterial substances such as lipopolysaccharide can be used to develop disease- or type-specific exosomes suitable for the resolution of chronic inflammation [146]. Lastly, surface modification of pullulan, which can bind to hepatocyte receptors, was shown to enhance the therapeutic effects of MSC exosomes by improving their targeting of injured organs [147].

For exosome-based therapy to be safe and successful for clinical applications, the biodistribution of the administered exosomes must be explored. Currently, there are various studies to evaluate the biodistribution of exosomes obtained from different sources and treated in different disease models. In mice with acute kidney injury, intravenously delivered MSC exosomes were detected from the site of injury after 5 h and were not detectable after 24 h [148]. In tumor-bearing mice, the accumulation of exosomes was significantly higher in the tumor area at 24 and 48 h following intraperitoneal injection [108]. In general, intravenously delivered exosomes are known to accumulate mainly in the liver and spleen, however, according to the cell source and route of administration, the biodistribution pattern can be different [149]. When designing a therapeutic strategy using exosomes, careful consideration for MSC source, administration route, and biodistribution must be preceded.

In conclusion, successful MSC exosome therapy of various rare diseases requires disease- and/or patient-customized therapy. To do so, detailed identification of the characteristics and pathogenesis of each target disease, selection of feasible exosome cargo, determination of methods to regulate selected factors, and improved delivery to lesions are required.

## Figures and Tables

**Table 1:** Ongoing clinical trials using MSC exosomes [23].

No.	Study Title	Conditions	Interventions	Locations	Last Update	Ref.
**1**	Effect of UMSCs Derived Exosomes on Dry Eye in Patients With cGVHD	Dry Eye	UMSC exosomes	Zhongshan Ophthalmic Center, Guangzhou, Guangdong, China	21 February 2020	NCT04213248
**2**	Effect of Microvesicles and Exosomes Therapy on β-cell Mass in Type I Diabetes Mellitus (T1DM)	Diabetes Mellitus Type 1	MSC exosomes	Sahel Teaching Hospital, Sahel, Cairo, Egypt	14 May 2014	NCT02138331
**3**	Evaluation of Adipose Derived Stem Cells Exo.in Treatment of Periodontitis	Periodontitis	AD-MSC exosomes	Beni-Suef University, Banī Suwayf, Egypt	17 February 2020	NCT04270006
				Cairo University, Cairo, Egypt		
**4**	Exosome of Mesenchymal Stem Cells for Multiple Organ Dysfuntion Syndrome After Surgical repair of Acute Type A aortic Dissection	Multiple Organ Failure	MSC exosomes	Fujian Medical University, Fujian, China	6 May 2020	NCT04356300
**5**	MSC-Exos Promote Healing of MHs	Macular Holes	MSC-Exo	Tianjin Medical University Hospital, Tianjin, China	17 September 2019	NCT03437759
**6**	MSC EVs in Dystrophic Epidermolysis Bullosa	Dystrophic Epidermolysis Bullosa	AGLE 102	Aegle therapetics, Miami, Florida, USA	12 January 2021	NCT04173650
**7**	The Use of Exosomes In Craniofacial Neuralgia	Neuralgia	Exosomes	Neurological Associates of West LA, Santa Monica, California, USA	6 January 2020	NCT04202783
**8**	Focused Ultrasound and Exosomes to Treat Depression, Anxiety, and Dementias	Refractory Depression	Exosomes	Neurological Associates of West LA, Santa Monica, California, USA	6 January 2020	NCT04202770
		Anxiety Disorders				
		Neurodegenerative Diseases				
**9**	The Safety and the Efficacy Evaluation of Allogenic Adipose MSC-Exos in Patients With Alzheimer’s Disease	Alzheimer Disease	MSCs-Exos administrated for nasal drip Dosage	Ruijin Hospital Shanghai Jiao Tong University School of Medicine, Shanghai, China	29 September 2020	NCT04388982
**10**	Allogenic Mesenchymal Stem Cell Derived Exosome in Patients With Acute Ischemic Stroke	Cerebrovascular Disorders	Exosomes	Saeed Oraei Yazdani, Tehran, Iran, Islamic Republic of	25 January 2021	NCT03384433
**11**	iExosomes in Treating Participants With Metastatic Pancreas Cancer With KrasG12D Mutation	Metastatic Pancreatic Adenocarcinoma	MSC exosomes with KRAS G12D siRNA	M D Anderson Cancer Center, Houston, Texas, United States	2 January 2020	NCT03608631
		Pancreatic Ductal Adenocarcinoma				
		Stage IV Pancreatic Cancer				
**12**	A Pilot Clinical Study on Inhalation of Mesenchymal Stem Cells Exosomes Treating Severe Novel Coronavirus Pneumonia	COVID-19	MSC exosomes	Ruijin Hospital Shanghai Jiao Tong University School of Medicine, Shanghai, China	7 September 2020	NCT04276987
**13**	A Tolerance Clinical Study on Aerosol Inhalation of Mesenchymal Stem Cells Exosomes In Healthy Volunteers	Healthy	MSC exosomes	Ruijin Hospital Shanghai Jiao Tong University School of Medicine, Shanghai, China	30 April 2020	NCT04313647
**14**	Evaluation of Safety and Efficiency of Method of Exosome Inhalation in SARS-CoV-2 Associated Pneumonia.	COVID-19	MSC exosome inhalation	Medical Centre Dinasty, Samara, Russian Federation	4 November 2020	NCT04491240
**15**	Organicell Flow for Patients With COVID-19	COVID-19	Organicell Flow	Landmark Hospital, Naples, Florida, United States	31 December 2020	NCT04384445
				Landmark Hospital, Athens, Georgia, United States		
**16**	Safety and Efficiency of Method of Exosome Inhalation in COVID-19 Associated Pneumonia (COVID-19EXO2)	COVID-19	MSC exosome inhalation	Medical Centre Dinasty, Samara, Russian Federation	26 October 2020	NCT04602442

## Data Availability

Not applicable.
